# An IDEA for Short Term Outbreak Projection: Nearcasting Using the Basic Reproduction Number

**DOI:** 10.1371/journal.pone.0083622

**Published:** 2013-12-31

**Authors:** David N. Fisman, Tanya S. Hauck, Ashleigh R. Tuite, Amy L. Greer

**Affiliations:** 1 The Dalla Lana School of Public Health, Faculty of Medicine, University of Toronto, Toronto, Ontario, Canada; 2 Department of Medicine, Faculty of Medicine, University of Toronto, Toronto, Ontario, Canada; 3 Department of Psychiatry, Faculty of Medicine, University of Toronto, Toronto, Ontario, Canada; 4 The Decision Centre for Infectious Disease Epidemiology (DeCIDE), Toronto, Ontario, Canada; 5 Modeling and Projection Section of the Professional Guidelines and Public Health Practice Division, Centre for Communicable Diseases and Infection Control, Public Health Agency of Canada, Toronto, Ontario, Canada; Department of Health, department of Physics, College of computer sciences, United States of America

## Abstract

**Background:**

Communicable disease outbreaks of novel or existing pathogens threaten human health around the globe. It would be desirable to rapidly characterize such outbreaks and develop accurate projections of their duration and cumulative size even when limited preliminary data are available. Here we develop a mathematical model to aid public health authorities in tracking the expansion and contraction of outbreaks with explicit representation of factors (other than population immunity) that may slow epidemic growth.

**Methodology:**

The Incidence Decay and Exponential Adjustment (IDEA) model is a parsimonious function that uses the basic reproduction number R_0_, along with a discounting factor to project the growth of outbreaks using only basic epidemiological information (e.g., daily incidence counts).

**Principal Findings:**

Compared to simulated data, IDEA provides highly accurate estimates of total size and duration for a given outbreak when R_0_ is low or moderate, and also identifies turning points or new waves. When tested with an outbreak of pandemic influenza A (H1N1), the model generates estimated incidence at the i+1^th^ serial interval using data from the i^th^ serial interval within an average of 20% of actual incidence.

**Conclusions and Significance:**

This model for communicable disease outbreaks provides rapid assessments of outbreak growth and public health interventions. Further evaluation in the context of real-world outbreaks will establish the utility of IDEA as a tool for front-line epidemiologists.

## Introduction

Outbreaks of novel emerging pathogens such as the SARS coronavirus [1], [2] or familiar pathogens whose characteristics have been changed by genetic shift or recombination events such as novel influenza virus strains [3], [4] are an important and ongoing global health concern [5], [6], [7]. While numerous factors contribute to pathogen emergence, including environmental change, global travel and commerce, and selective pressure via food production [5], [8], [9] public health authorities at regional, national, and international levels are confronted with the practical task of outbreak management and control. The capacity to describe the characteristics of epidemic processes in real time, predict the duration and size of epidemics, and quantify the transmission characteristics of new or mutated pathogens poses a major challenge to public health professionals.

Mathematical models provide a useful framework for characterization and quantification of ecological processes, including outbreaks of infectious diseases [10], [11]. However, typical model forms focus largely on the epidemiological characteristics of the disease at the time of emergence, and while such models may be used as a platform for projection of intervention effects, they seldom explicitly account for the behavioral, regulatory, and informational interventions that are either put into place by public health authorities, or occur spontaneously in a worried public, once knowledge of an epidemic is widespread [12]. Such limitations can be overcome post-hoc through model fitting and calibration, but insights from models would be most helpful early in outbreaks and epidemics, when such data is almost uniformly unavailable. Standard mathematical models that attempt to project outbreak duration and final size based on initial characteristics will predictably overestimate final outbreak size, since reproduction numbers decline not only due to depletion of susceptible individuals, but also to spontaneous and planned control activities and behaviours [1], [13], [14].

Here we propose a simple phenomenological model derived from observations that estimates of the basic reproduction number R_0_ fail to accurately project the contours of outbreaks when control interventions are put into place, and in a manner that cannot be attributed simply to misspecification of depletion of susceptible individuals. We propose that this simple model could find application early in the course of an outbreak for provision of credible and easily interpreted projections on outbreak timing, control, and final size.

## Methods

### Model development

The study was approved by the Research Ethics Board, University of Toronto. The Incidence Decay and Exponential Adjustment (IDEA) model is based on concept of the basic reproduction number, R_0_, defined by Vynnycky and White as “the (average) number of successful transmissions per infected person” [11] when an infected person first enters a completely susceptible population [11], [15]. The rate of growth of an epidemic is a function of both R_0_ and the average serial interval, which is defined as the time between symptoms developing in an index case and symptoms developing in a secondary case [11], [15]. We use a symptom-based interval so that the IDEA model is applicable in situations where microbiological or serological diagnosis is not available. Early in an outbreak or epidemic, incident case counts (*I*) in each serial interval *t* may be defined as:
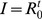
(1.0)


The basic reproduction number thus describes initial exponential growth of an outbreak or epidemic. As this process continues, the effective reproduction number *R* is often defined as *R_0_ x S/N*, where *S/N* is the proportion of the population that remains susceptible to infection (defined as S  =  number of susceptibles divided by N  =  total population size), and the decline in *R* with time results in ultimate termination of the epidemic. However, many outbreaks rapidly dampen after a short period of time, in a manner that cannot be attributed to a decline in susceptibles [1], [13], [14], [16]. A potential mechanism driving decline in epidemics in the presence of susceptibles is spontaneous or planned reduction in the components of R_0_ itself (disease duration, contact rate, and infectiousness of cases) either because of public health interventions, or due to concern about disease among members of the public. As a decline in *R* for this reason is unlikely to be estimable in real time in the context of an outbreak, we propose that control be modeled empirically in a time-varying manner analogous to a financial discounting function. The impact of this discounting or dampening factor on case counts may be expressed empirically as:
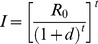
(2.0)where *d* is a discount factor. The model may be fitted to available outbreak data when case counts are aggregated to reflect likely generation times, and can be readily calculated using information on latent and infectious periods, which are available for many pathogens of public health importance [17], or estimated empirically for novel pathogens [1], [18].

### Model Properties

In order to more fully understand the model's performance based on varying disease and disease control characteristics, we created a difference equation model with discrete time steps, each representing a single disease generation. The model was specified as follows:
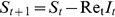
(3.0)

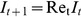
(3.1)

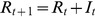
(3.2)Here S is the number of susceptibles in the population, I is the number of infectives, and R is the number of immune individuals. The total population size N  =  S+I+R. Re_t_ is the time varying effective reproductive number: the number of new infectious cases in a given generation created by each infective individual in the last generation. Re_t_ is a function of the basic reproductive number, R_0_. Typically, Re_t_ is expressed as R_0_S_t_/N but such a formulation fails to account for control activities and dynamic changes in population behavior that may reduce transmissibility of infection.

We defined Re_t_ as: Re_t_  =  R_0_ κ_t_ S_t_/N where κ is a function of time and represents the proportionate reduction of risk of transmission via control activities. κ_t_ is defined as the relative risk of disease transmission (RR) raised to some power, such that κ_t_  =  RR^x^. Here x is some exponential function of t such that x  =  t^n^ and n is an integer > = 0. We refer to *n* as the “order” of control. For 0^th^ order control, the impact of control does not change over time, and Re is simply reduced by a constant fraction throughout the epidemic. For first order control, disease risk is reduced in a manner that accelerates with time; second and third order control represent “accelerating acceleration of control”, and so on.

We used this simple difference equation model to evaluate the fit of the IDEA model to simulated epidemics under different assumptions about infectiousness (R_0_), varying orders of control, under-reporting of cases, and multiple waves of infection. Models were fit by minimizing root-mean-squared differences (RMSD) between generation-specific case counts by adjustment of the R_0_ and d parameters of the IDEA model. When evaluating the performance of the IDEA model as applied to an SIR difference model under different assumptions about the order of κ, we normalized RMSD by dividing by total case counts, as higher order control resulted in smaller epidemics (and consequently smaller RMSD).

In addition to generating empirical estimates of *R_0_* and *d* parameters via fitting, the model can be manipulated algebraically to generate estimates of t_max_, the generation where the number of new cases is <1, such that the outbreak is effectively over. Multiplication of t_max_ by serial interval duration in calendar time provides an approximate estimate of outbreak duration. By manipulating [2.0] it can be seen that:
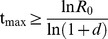
(4.0)


Integration of [2.0] over *t* also provides a complex expression which predicts total outbreak size, such that:
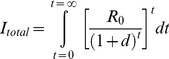
(4.1)

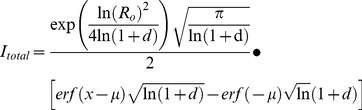
(4.1.1)Where
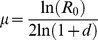
(4.1.2)Here *erf* is the so-called “error function”.

### Model Application

To test the ability of the model to describe simple epidemic dynamics in an actual outbreak, we applied the model to an outbreak of pandemic influenza A (H1N1) from the territory of Nunavut, Canada, using an empirically derived serial interval of 5 days [18]. Serial intervals may not be constant throughout outbreaks [19], [20] but are assumed to be nearly constant for the purposes of this model. The model was fitted to empirical case counts by minimizing sum-of-squares differences between model-derived and empirical case counts.

We obtained the daily number of laboratory-confirmed cases of pandemic H1N1 influenza (in which the cases were reported based on the earliest date of symptom onset, initial care, specimen collection, hospital admission, or ICU admission) for each community under study. A laboratory-confirmed case was reported as an individual with influenza-like illness or severe respiratory illness who tested positive for pandemic H1N1 influenza A virus by real-time reverse-transcriptase PCR (RT-PCR) or viral culture as is typical for Canadian influenza surveillance. As such, cases likely represent a subset of total true influenza cases [21], [22]. These data were provided by the Nunavut Department of Health and Social Services (HSS) and their use in this study was been approved by the Nunavut Chief Medical Officer of Health (Dr. Geraldine Osborne) and Michael Ruta (Territorial Epidemiologist) in 2009. No identifying data regarding individual cases was shared with the research team or used for subsequent analyses. All data included in the dataset used for model evaluation were aggregate, daily case counts for de-identified Nunavut communities. As a result, these data were not deemed protected health information by the territory of Nunavut and therefore, no patient consent was deemed necessary.

Cases were normalized to the first day of the outbreak (day 1). The definition of an outbreak was based on the Ontario Ministry of Health and Long Term Care (MOHLTC) guidelines [23]. In this instance, two cases are considered unrelated if they are separated by more than the sum of the incubation period and the period of communicability for the causative agent, which is 6 days for pandemic influenza A (H1N1). Data points which could not be considered part of the same outbreak (more than 6 days apart) and all outbreaks less than three serial intervals (15 days) were excluded from the analysis. It was assumed that short outbreaks (less than 15 days for pandemic influenza A (H1N1)) would essentially be over by the time an effective and intensive public health response was mobilized.

Simulations were performed using the Berkeley Madonna dynamic systems modeling package (University of California, Berkeley; http://www.berkeleymadonna.com), and model fits for Nunavut data were performed using the “Solver” application for Microsoft Excel (Frontline Systems, Incline Village, Nevada; http://www.solver.com).

## Results

### Simulations

**Figure 1:**
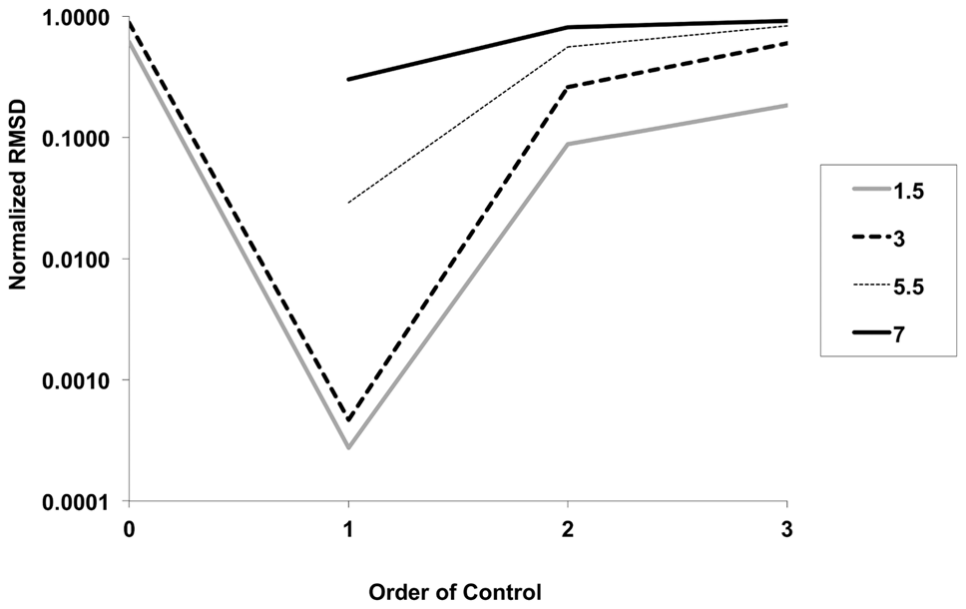
Model fits and “order of control”. Relationship between final-size-normalized root-mean squared differences (RMSD, Y-axis) between SIR model outputs and IDEA model fits, for R_0_ ranging from 1.5 to 7 (legend), with variation in order of control in SIR models (X-axis). It can be seen that for all R_0_ best-fits are achieved with first order control. Model fits were however better with low R_0_ simulations than with higher R_0_ simulations.

Normalized sum of squares fits of the IDEA model to simulated data were best with first order control (i.e., κ  =  RR^t^), and were better for systems with low or moderate R_0_ (i.e., R_0_< = 5) than those with higher R_0_ (**Figure 1**). Model projections of final epidemic sizes were extremely accurate for a range of R_0_ values; however, as R_0_ increased beyond 5.5, model projected end dates for epidemics were later than those seen in simulated data (**Figure A in File S1**).

**Figure 2:**
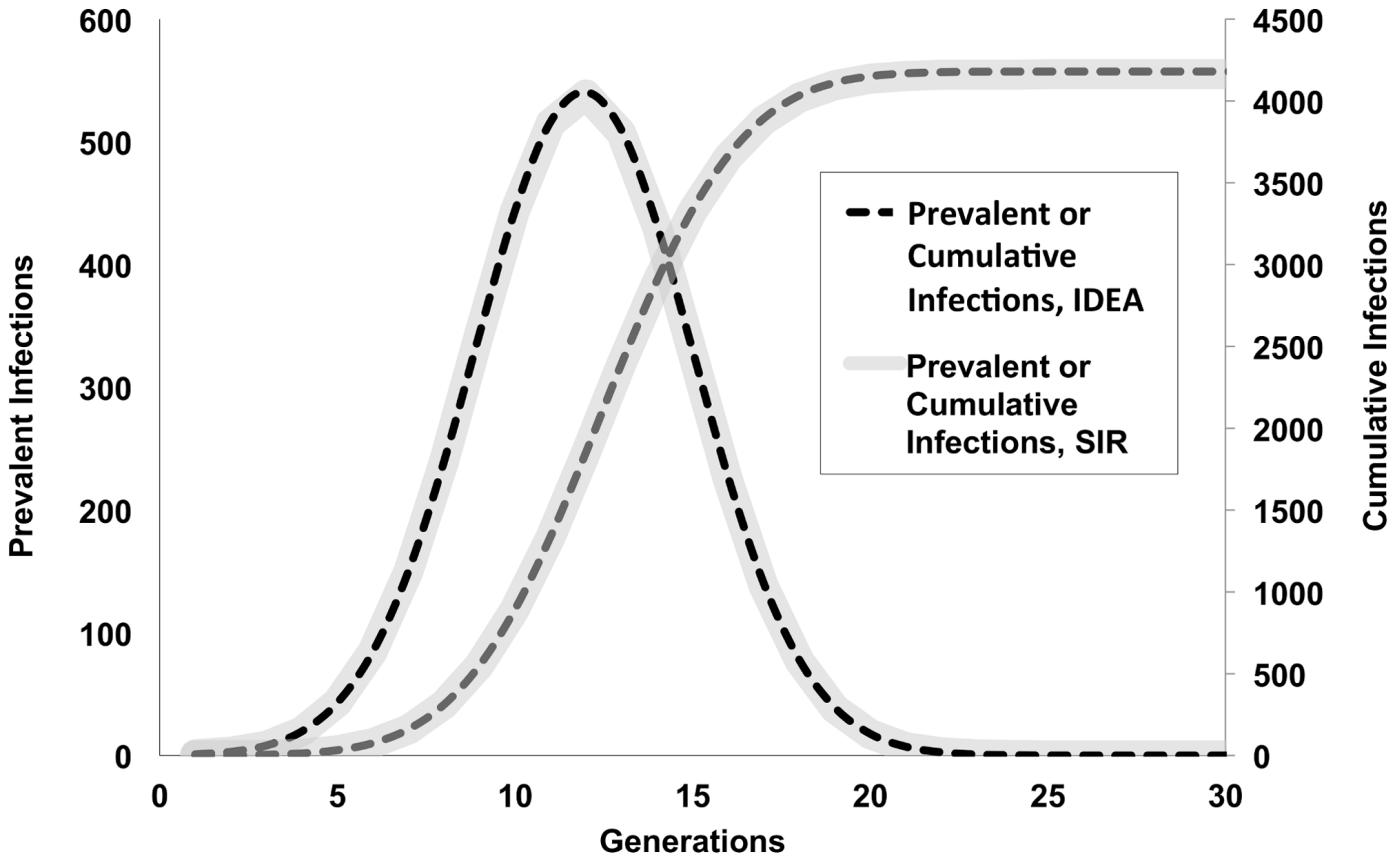
IDEA model fits for low R_0_ epidemics. Comparison of prevalent infections and cumulative infections from data generated using the SIR difference equation model described in the text (gray curves), and an IDEA model fitted to the first four generations of the simulated SIR epidemic (dashed curves). The true R_0_ used in the SIR model was 3.0. It can be seen that the IDEA model projections reproduce future case counts in the SIR model almost perfectly.

For systems with low or moderate R_0_, and assuming first order control, stable parameters were identified for the IDEA model within 3–4 generations, and the use of these parameter values accurately projected the full extent of the epidemic curve (**Figure 2**) in a manner that made IDEA model projections and simulated data indistinguishable. Empiric best-fit values for the “discount parameter” d were approximately 0.054 or 0.055 for all low or moderate R_0_ models. Best-fit R_0_ values identified for the IDEA model tended to be slightly higher than true R_0_ values, and the proportionate degree of over-estimation increased as the true R_0_ increased.

**Figure 3:**
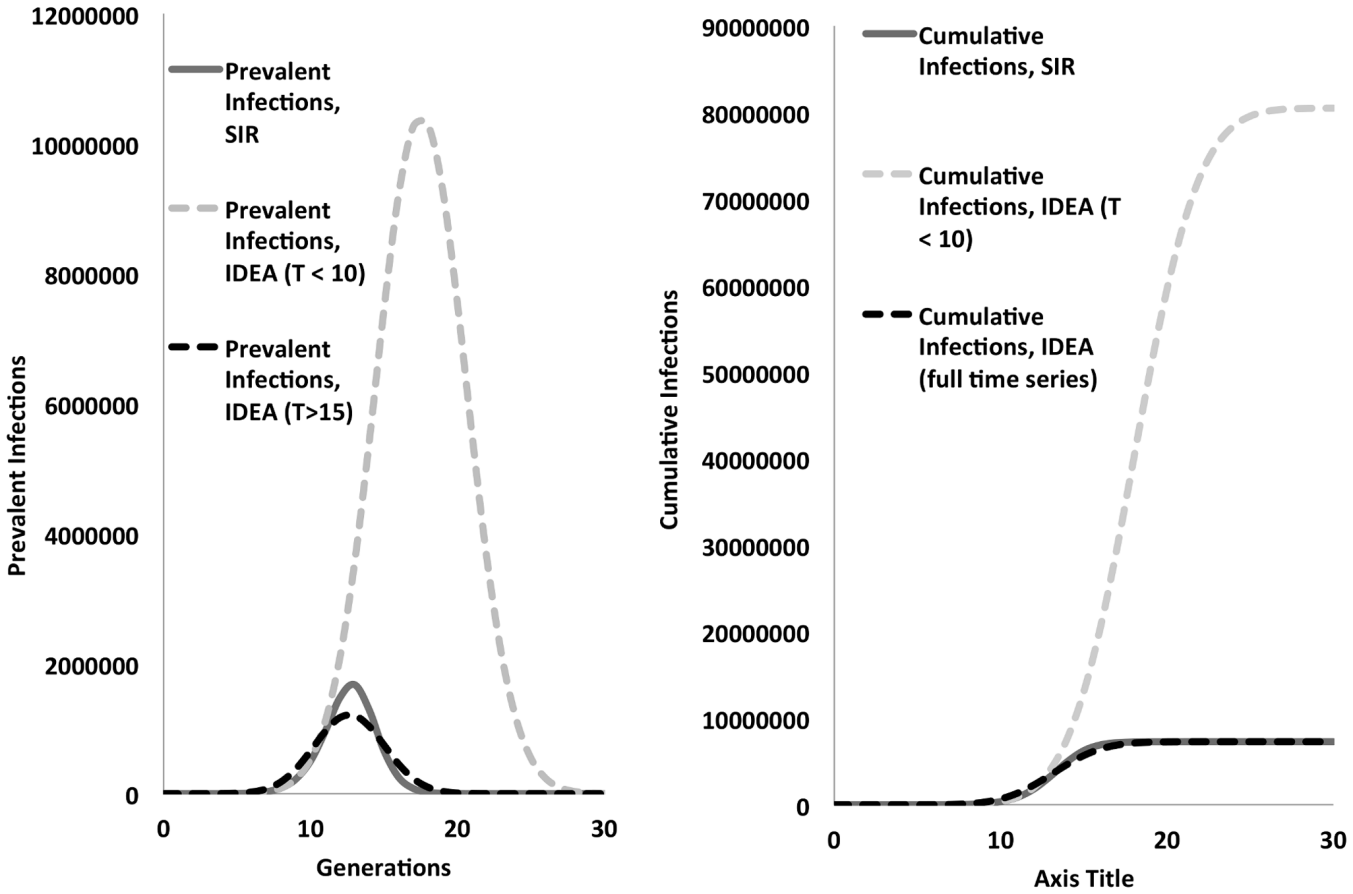
IDEA model fits for higher R_0_ epidemics. Concordance between simulated data from an SIR difference model for a higher-R_0_ system (R_0_ = 6) (solid gray curves) and IDEA fits based on early (T < = 10) generations (gray dashed curves), and based on fits from generation 15 onwards (black dashed curves). Prevalent infections are shown in the left hand panel while cumulative infections are shown on the right. Fits from generations prior to the epidemic peak (T< = 10) reproduce the initial growth of the epidemic well, and also provide accurate estimates of the true R_0_ (R_0_∼6.34, d = 0.054); however, these parameters result in IDEA projections of far larger epidemics than actually occur. Once IDEA models are fit using generations that include and follow the epidemic peak (i.e., T> = 15) projections of both prevalent and cumulative infections become fairly accurate (black dashed curves); however, estimated R_0_ is much larger than the true value (R_0_∼7.56) and the best-fit value for d increases as well (from 0.054 to 0.069).

In simulated epidemics with high R_0_ initial convergence occurred rapidly as the epidemic grew, with best-fit values of d approximately 0.054 or 0.055, and accurate estimation of true R_0_ values, in approximately 4 generations. However as the simulated epidemic peak occurred, best-fit R_0_ estimates, and d estimates for the IDEA model both increased sharply diverging from initial estimates and, allowing the IDEA model to reproduce epidemic peaks and subsequent declines (**Figure 3** and **Figures B and C in File S1**). For high R_0_ systems, R_0_ estimates obtained via fitting after the epidemic had peaked were far higher than true R_0_ values and than values estimated prior to peaks.

Under-reporting of cases is expected to occur for a variety of diseases of public health importance; we evaluated IDEA fits to SIR model outputs where increasing fractions of cases were unobserved and consequently unavailable for fitting. In fact, we found parameter estimates and final-size-normalized RMSD model fits to be quite stable as long as case reporting fractions exceeded 5% (**Figures D, E, and F in File S1**); IDEA model fits became unstable only with low absolute numbers of reported cases. We evaluated the impact of multi-wave epidemics on IDEA model fitting, and found that while the structure of the IDEA model made it difficult to fit to multi-wave epidemics, an important indicator of the emergence of a new wave of infection was an increasing gap between sequential best-fit values of the discount factor d as time series used for fitting were extended to include additional waves (**Figure G in File S1**). We term this indicator Δ*d*, such that
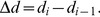
(4.2)


Many outbreaks are characterized by sequential “waves” that may either signify the impact of seasonal or behavioural influences on disease transmission [24], signify the movement of epidemics into previously unaffected sub-populations [25], or (as in the case of SARS in Canada) may signify failure of control measures [26].

**Figure 4:**
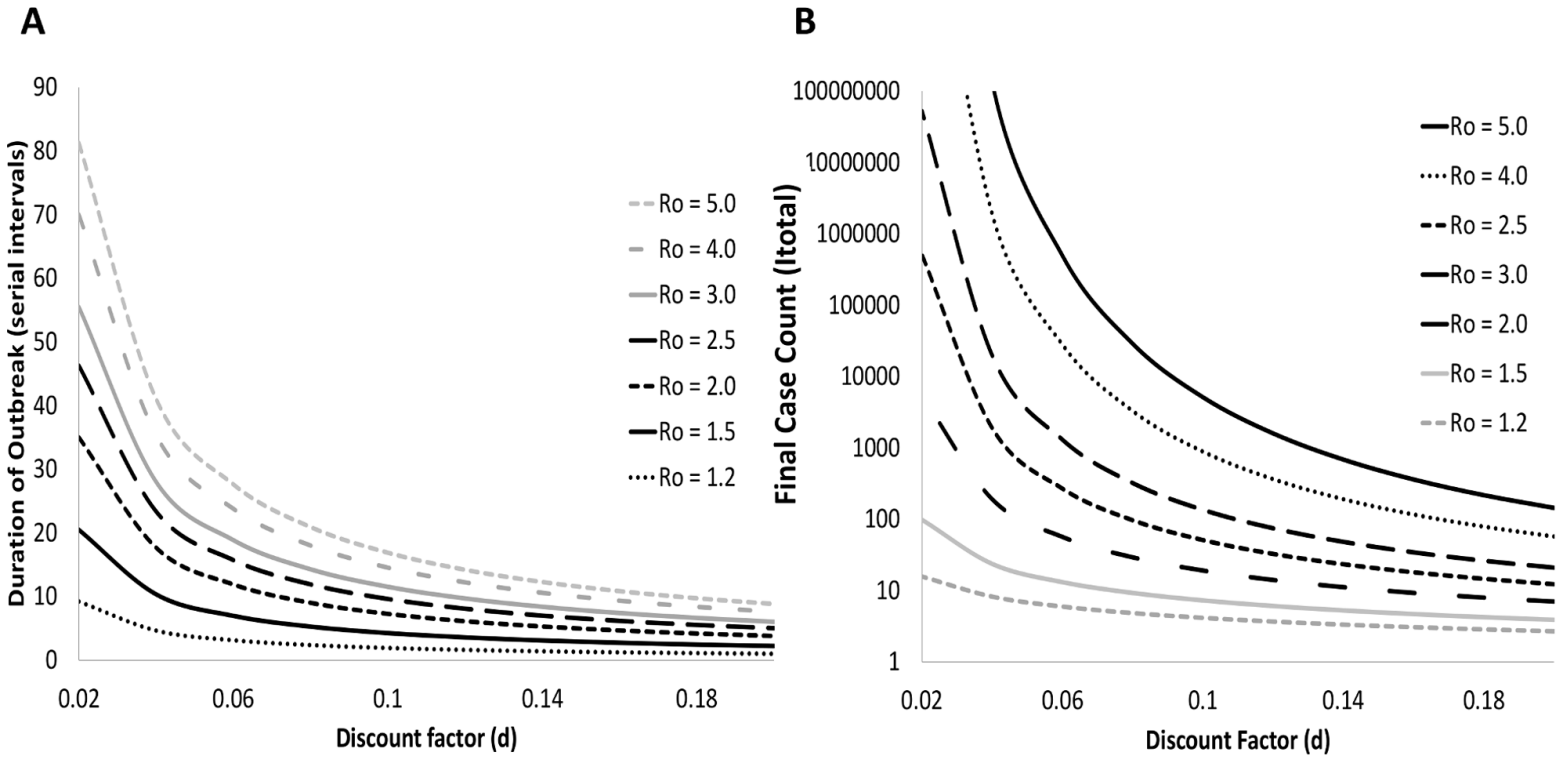
Model behaviour. The overall behaviour of the IDEA model based on a range of possible R_o_ and d values (a) the variation of t_max_ or outbreak duration as a function of R_o_ and d (b) the variation of I_total_ or the final cumulative incidence as a function of R_0_ and d.

As the IDEA model appeared to provide a reasonable means of modeling epidemics, especially for R_0_< = 5, we evaluated the expected relationship between R0, d, tmax and Itotal mathematically, using formulae 4.0 and 4.1 for a range of possible R0 and d values. The IDEA model generates an estimate of R0 and d at each point in an outbreak, and it is then possible to rapidly project the estimated duration and total cases of the outbreak. These results are presented graphically in **Figure 4**.

### Application

**Figure 5:**
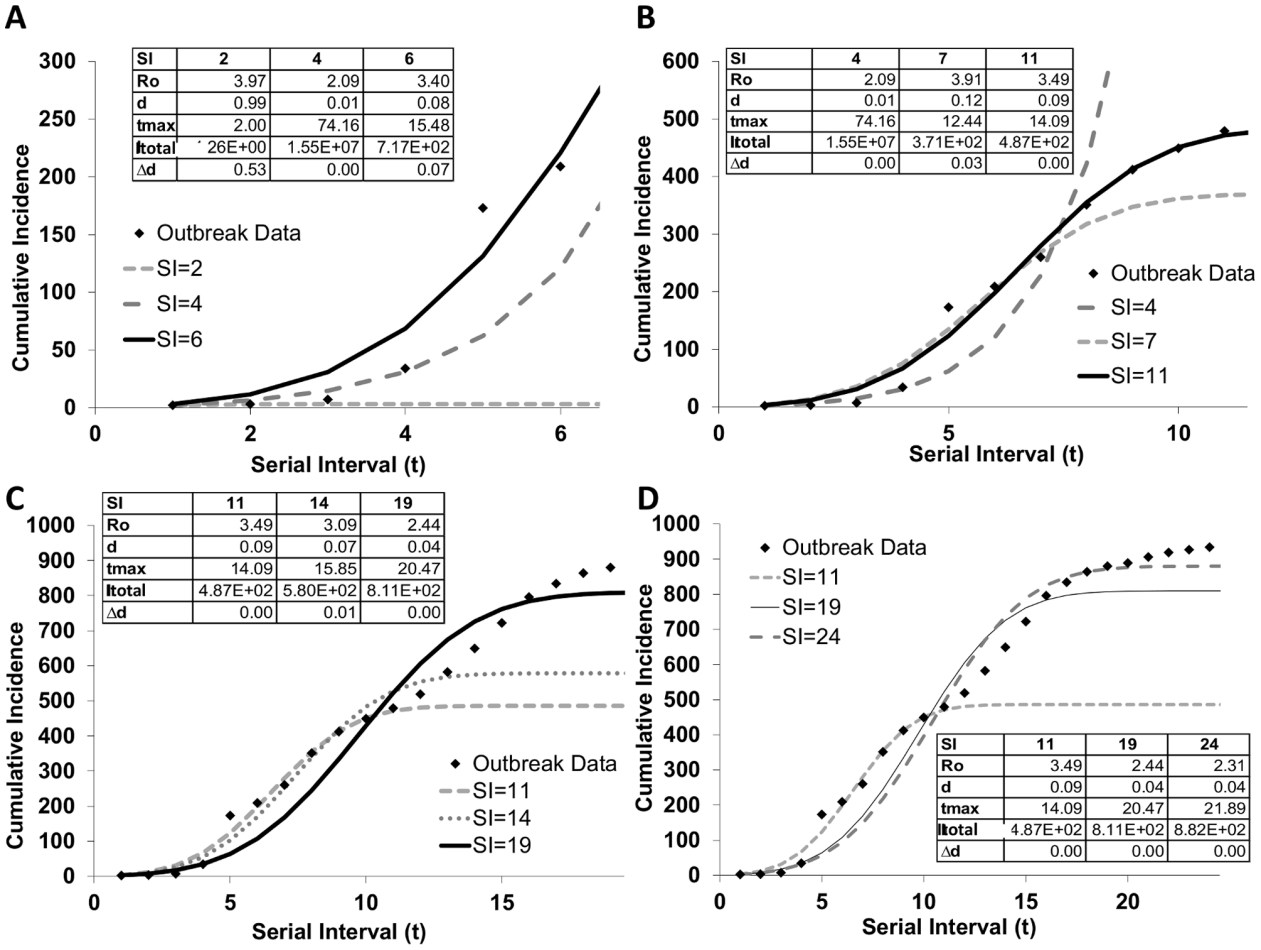
Pandemic H1N1 case counts modeled with the IDEA Model. The IDEA model applied to an outbreak of influenza A (H1N1) in Nunavut, Canada, with the model parameters R_0_, d, t_max_, I_total_ and Δd. (a) the early stages of the outbreak, with largely exponential growth, (b) dampened growth with reduced projected t_max_ values by serial interval 7, (c) a second wave in the outbreak and (d) the fit of the model at 24 out of 27 generations.

The Nunavut, Canada data illustrate the behaviour of the model in a real outbreak situation (**Figure 5**). This outbreak took place over 27 serial intervals and included 950 cases. The population of Nunavut in 2011 was 31,906 [27]. Initially, the outbreak is unable to gain momentum, as shown in **Figure 5a** by the curve predicted when two serial intervals of data are known (SI = 2). Once four serial intervals are known, however, the outbreak grows exponentially and the model (SI = 4) projects a t_max_, or outbreak duration, of 74 serial intervals. By SI = 6 (the model fit with 6 serial intervals), the projected t_max_ is drastically dampened to 15 generations. In these early stages of the outbreak, the IDEA model is able to rapidly determine whether the outbreak is growing or stabilizing, based on the change in t_max_ and the change in Δd.

In later stages of the outbreak (shown in **Figures 5b, c and d**), the model continues to provide a rapid analysis of the immediate direction of the outbreak. **Figure 5c** shows a key inflection point at which the outbreak suddenly began growing again. Such biphasic outbreaks can occur for various reasons, such as the end of school closure periods, the arrival of a newly infected individual into a community or a reduction in a public health intervention such as hand sanitizer provision. The model illustrates the new projection in the outbreak behavior as such events occur.

**Figure 6:**
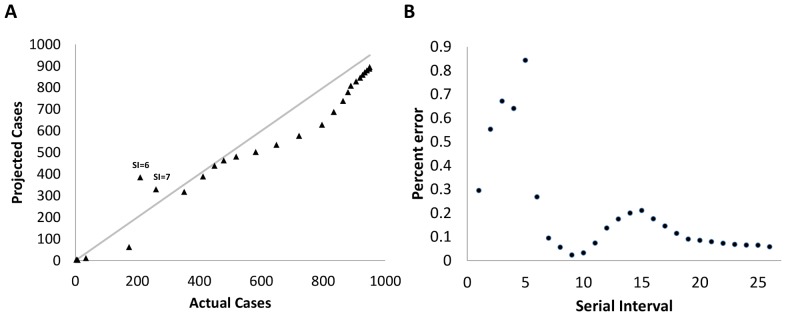
Utility of the IDEA model in evaluating the impact of public health and social or environmental factors on outbreak behaviour. (a) The utility of the IDEA model in evaluating the level of control over the outbreak. Each projection is based on the outbreak up to i intervals, projected to the i+1^th^ interval. With the exception of serial intervals 6 and 7 illustrated in the figure, the projected case counts were less than the actual case counts implying that at each serial interval the outbreak grew more than would be expected by its previous course. During this outbreak, the model underestimated the actual number of cases except during two serial intervals. (b) Percent error between the projection for the next generation and actual case counts according to generation.

Estimating the impact of public health interactions and the degree of control over an outbreak is a considerable challenge while an outbreak is ongoing. As a result, the IDEA model was used to compare actual versus projected cases as a means of judging whether the outbreak was under control. **Figure 6a** shows the Nunavut outbreak with the actual cases of influenza on the × axis and the cases projected by IDEA model on the y axis. Each point on the y axis represents the model fitted to *i* generations and applied to *i+1* generations. On average, projections to the next generation are correct within 20.3% [95% credible interval 11.8, 28.8]. In **Figure 6a** we propose that when y>x, the model is projecting excess cases, implying that at this snapshot in the outbreak, the current generation had slowed its growth. Similarly, as the outbreak shifts to the y<x side of the line, the model is projecting a total case count lower than the actual outbreak, implying that the outbreak is uncontrolled and that the current generation has exceeded the model's projection. **Figure 6b** shows the trend in percent error as the outbreak progresses and demonstrates that after the seventh serial interval the percent error remains below 22%.

## Discussion

With the development of the IDEA model, we have demonstrated a simple, versatile model for emerging communicable disease outbreaks that has the capacity to provide short term projections of outbreak growth and contraction. To the best of our knowledge, this is the first application of this particular descriptor to epidemic growth, though other fitting methods of varying complexity are well described [25], [28], [29], [30]. However, Wu and Huberman have previously described an approach similar to that outlined above to describe the growth and decay of interest in news items on the Internet, with exponential growth countered by a “discount factor” that damps the “reproductive number” for page sharing as a function of time [31]. We found that best-fit projections for the IDEA model for disease dynamic systems with low or intermediate R_0_ were exceedingly good, with parameters derived within 3–4 generations able to project the full extent of simulated epidemics with remarkable accuracy. If validated, the implications of such a finding may be profound (e.g., the ability to project, with a high degree of accuracy, the final size and duration of a seasonal influenza outbreak within 2 weeks of onset).

The application of the model to simulated epidemics with higher R_0_ (>5) was more challenging, as best-fit parameters derived from early outbreak generations, while close to true R_0_ values, resulted in epidemic curves that dramatically overshot true epidemics (a difficulty similar to that often encountered when attempting to fit an SIR model to early outbreak data). Nonetheless, the application of this technique to high R_0_ epidemics may be useful for a variety of reasons: first, early (pre-epidemic peak) IDEA estimates of R_0_ closely matched true R_0_ values in simulations, suggesting that the use of this technique for early R_0_ estimation when novel diseases emerge may be reasonable regardless of whether R_0_ is low or high. Furthermore, the Δd metric, and the abrupt shift in R_0_ estimates that occurs with the epidemic peak would provide a helpful signal to epidemiologists that the epidemic is peaking or changing. Finally, as parameter estimates stabilize again for high R_0_ systems, the IDEA model remains a useful tool for projecting the total size and duration of an outbreak. It is also possible that challenges in fitting the IDEA model to simulated data represent not a limitation of the IDEA model, but are rather an artefact of our use of SIR difference equation models, which tend to peak and collapse suddenly with at high R_0_.

The utility of this model was evaluated further with data from a large outbreak of pandemic influenza A (H1N1) and the potential of the IDEA model to begin to understand the impact of public health interventions and structural and human behavioural factors in outbreaks was also explored. Although the IDEA model can provide no hypothesis about which factors caused a sudden acceleration or deceleration of the outbreak, it provides a fast barometer of the situation, based on all known cases.

Further testing and development in real-time outbreak situations will be needed before the IDEA model can be used in public health interventions for nearcasting (short term outbreak projection) and to assess the impact of public health interventions and to separate the impact of such interventions from spontaneous behavioural changes. The model's main asset is its simplicity and the fact that it does not require consideration of population immune status for parameterization. The model is constructed entirely on a case count time series that is likely to be available to public health professionals charged with outbreak control. IDEA requires no sophisticated knowledge of mathematics or computing, and can be realized using commonly available spreadsheet programs. The model's outputs, which include both cumulative case counts under best-fit conditions, and cumulative outbreak duration, would be valuable to front-line public health professionals seeking to budget material and human resources needed to see an outbreak through to its conclusion. This simplicity may make the model especially useful in resource-limited settings where rapid assessment of both outbreak behaviour, and *change* in outbreak behaviour is needed.

Nevertheless, the simplicity of the IDEA model is also a limitation, as it cannot provide insight into the fundamental workings of outbreaks. The factors driving contraction of growth are non-specific and could include the impact of public health interventions, changes in population behaviour, saturation of sub-populations with infection, and changes in the physical environment that speed or slow epidemic spread (e.g., rainfall or change of season).

In situations where limited public health resources must be allocated to one region at the expense of another, this model may aid in deciding which region is experiencing an outbreak that is growing more rapidly, and which region has stabilized, while using minimal data. Moreover, the model may aid in the assessment of public health interventions. If a drastic intervention is implemented, such as the closing of schools, the model may be able to rapidly identify (by means of a sudden reduction in the expected length of the outbreak t_max_) that the intervention is having a positive impact on slowing the outbreak.

Our application of this simple model to influenza outbreak data in an isolated Canadian population has been encouraging, and it is our hope that other groups will assess the usefulness of this model in the context of other diseases and demographic groups. We also hope to translate knowledge regarding this model to front-line public health professionals who may be able to assess its usefulness in real-time. Given the ceaseless emergence of novel communicable disease threats that challenge current public health professionals, we expect no shortage of opportunities for such applications.

## Supporting Information

File S1 **Combined file of supporting figures. Figure A: IDEA estimates of total epidemic size and duration.** The figure plots percent deviation of the IDEA model from simulated epidemic size data (gray dashed curve) and epidemic duration data (black dashed curve) with increasing basic reproductive number (R_0_). Across a broad range of values of R_0_, final size estimates from the IDEA model remained accurate. However, when R_0_ exceeded a threshold of ∼6, there was an increasing tendency for the IDEA model to project the epidemic to end later than was in fact the case. This may represent a limitation of the IDEA model, but may also be an artifact of the sudden “collapse” of epidemics with high R_0_ in SIR simulations. **Figure B: IDEA estimates of R_0_ and d by generations of data available.** Estimated values of R_0_ derived via IDEA model fits, according to generations of data available, with varying R_0_, from SIR model simulations with first order control. True R_0_ values are presented in the legend; fitted R_0_ estimates are presented on the Y-axis. It can be seen for R_0_< = 5, best-fit R_0_ values and true R_0_ values agree closely. High R_0_ models demonstrate similar concordance prior to epidemic peaks (which occur for high R_0_ models in generations highlighted by the shaded rectangle). However, in order to reproduce peaks and subsequent declines, IDEA model fits to simulated epidemic curves required higher R_0_ values than true R_0_ values, or R_0_ estimates obtained prior to the epidemic peak. **Figure C: IDEA estimates of R_0_ and d by generations of data available.** Estimated values of the “discount factor” d derived via IDEA model fits, according to generations of data available, with varying R_0_, from SIR model simulations with first order control. True R_0_ values are presented in the legend; Estimates of d are presented on the Y-axis. It can be seen for R_0_ < = 5, d stabilizes with a value of around 0.054, in fewer than 5 generations and remains stable. High R_0_ models demonstrate similar stability in d (and empiric values of d) prior to epidemic peaks (which occur for high R_0_ models in generations highlighted by the shaded rectangle). However, in order to reproduce peaks and subsequent declines, IDEA model fits to simulated epidemic curves required extremely high d values; the greater the true R_0_ the higher the value of d required to reproduce the epidemic curve in its totality. **Figure D: Impact of under-reporting.** Impact of under-reporting of cases generated using SIR difference model on IDEA model fits, as assessed with final-size-normalized root-mean-squared differences (RMSD). It can be seen that over a range of simulated R_0_ utilized in the SIR model, fits remained good except where under-reporting resulted in extremely small absolute case numbers. This is reflected in the fact that low-R_0_ fits are more sensitive to under-reporting than high R_0_ fits. The legend presents R_0_ values used in SIR models. **Figure E: Impact of under-reporting.** Impact of under-reporting of cases generated using SIR difference model on best-fit estimates of the discount factor d generated using the IDEA model. Best-fit values of d, based on a complete 30 generation time series, are robust in the face of a wide range of under-reporting but become unstable when very small absolute numbers of cases are reported. **Figure F: Impact of under-reporting.** Impact of under-reporting of cases generated using SIR difference model on best-fit estimates of R_0_ generated using the IDEA model. Best-fit values of R_0_ values are fairly stable; notably, as under-reporting increases, the best estimate of R_0_ for the high-R_0_ SIR outputs actually becomes a progressively better approximation of the true R_0_. **Figure G: IDEA fits to biphasic epidemic.** A biphasic epidemic was simulated using the SIR difference model with an R_0_ of 3, as described in the text (solid black curve). IDEA model fits, based on early generations (pale gray curve, for generations up to generation 16, prior to the onset of the second wave) and on all generations up to and including the peak of the second wave (dashed curve) are superimposed on the biphasic epidemic curve. The IDEA model's structure makes fitting to multiple peaks impossible; the best-fit IDEA model is based on parameters that create a single peak epidemic with a duration similar to that seen with the biphasic epidemic. **Figure H: IDEA fits to biphasic epidemic.** Best estimates of R_0_ and d for a biphasic epidemic, according to the number of generations available for model fitting. It can be seen that fits are perturbed by the onset of a second peak. The difference in d between sequential fits increases with the second wave (denoted by the shaded area), such that increases in this delta d parameter represent a potentially useful indicator of the onset of a second epidemic wave.
(PPTX)
