# Supplementary material for: An IDEA for Short Term Outbreak Projection: Nearcasting Using the Basic Reproduction Number
Source: PLoS One. 2013 Dec 31;8(12):e83622. doi: 10.1371/journal.pone.0083622 (PMC3877403; doi:10.1371/journal.pone.0083622)
Supplement: File S1 — Combined file of supporting figures. Figure A: IDEA estimates of total epidemic size and duration. The figure plots percent deviation of the IDEA model from simulated epidemic size data (gray dashed curve) and epidemic duration data (black dashed curve) with increasing basic reproductive number (R0). Across a broad range of values of R0, final size estimates from the IDEA model remained accurate. However, when R0 exceeded a threshold of ∼6, there was an increasing tendency for the IDEA model to project the epidemic to end later than was in fact the case. This may represent a limitation of the IDEA model, but may also be an artifact of the sudden “collapse” of epidemics with high R0 in SIR simulations. Figure B: IDEA estimates of R0 and d by generations of data available. Estimated values of R0 derived via IDEA model fits, according to generations of data available, with varying R0, from SIR model simulations with first order control. True R0 values are presented in the legend; fitted R0 estimates are presented on the Y-axis. It can be seen for R0< = 5, best-fit R0 values and true R0 values agree closely. High R0 models demonstrate similar concordance prior to epidemic peaks (which occur for high R0 models in generations highlighted by the shaded rectangle). However, in order to reproduce peaks and subsequent declines, IDEA model fits to simulated epidemic curves required higher R0 values than true R0 values, or R0 estimates obtained prior to the epidemic peak. Figure C: IDEA estimates of R0 and d by generations of data available. Estimated values of the “discount factor” d derived via IDEA model fits, according to generations of data available, with varying R0, from SIR model simulations with first order control. True R0 values are presented in the legend; Estimates of d are presented on the Y-axis. It can be seen for R0 < = 5, d stabilizes with a value of around 0.054, in fewer than 5 generations and remains stable. High R0 models demonstrate similar stability in d [file pone.0083622.s001.pptx]

## Slide 1
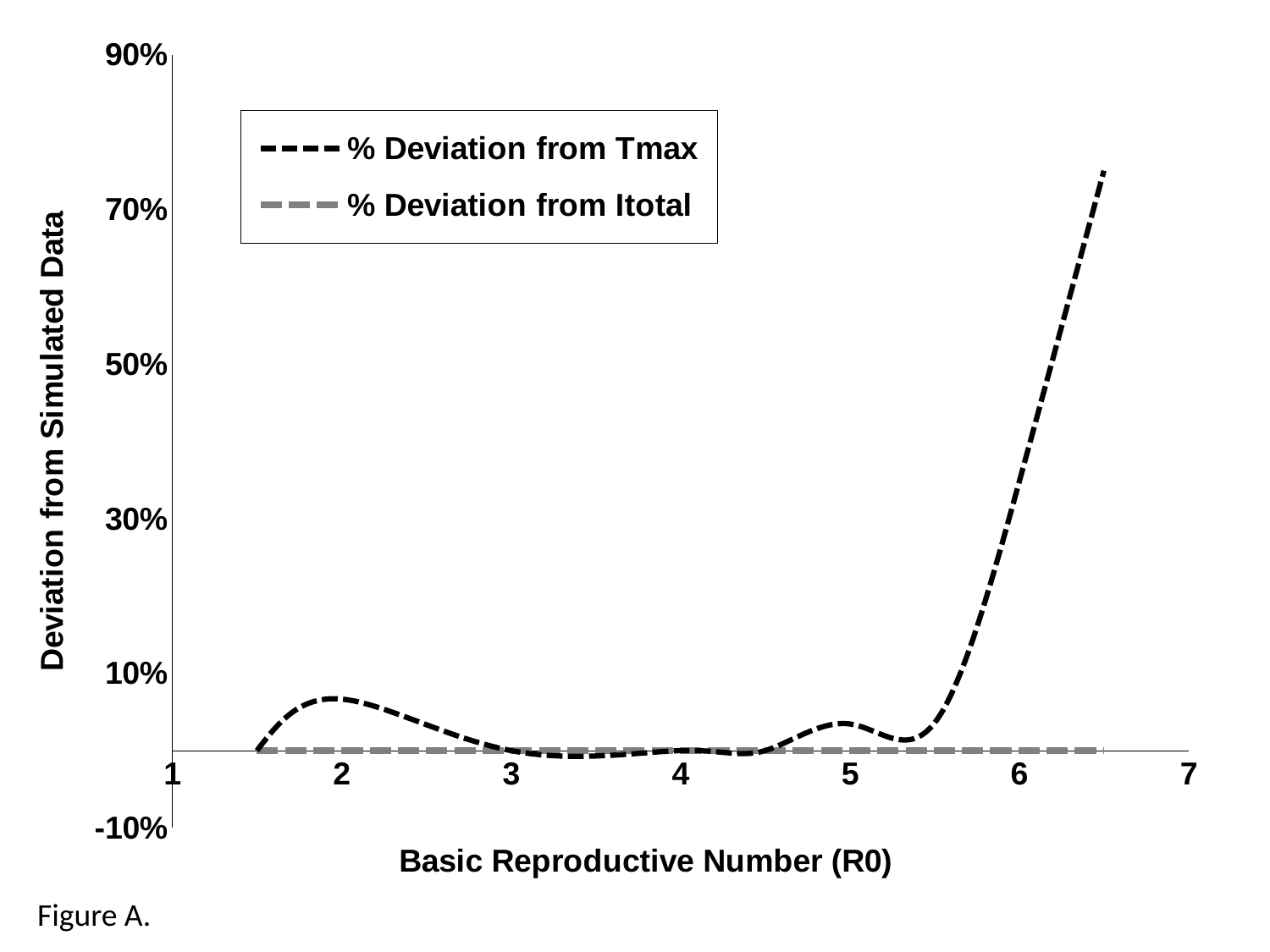

### Chart
| Category | % Deviation from Tmax | % Deviation from Itotal |
|---|---|---|Figure A.

## Slide 2
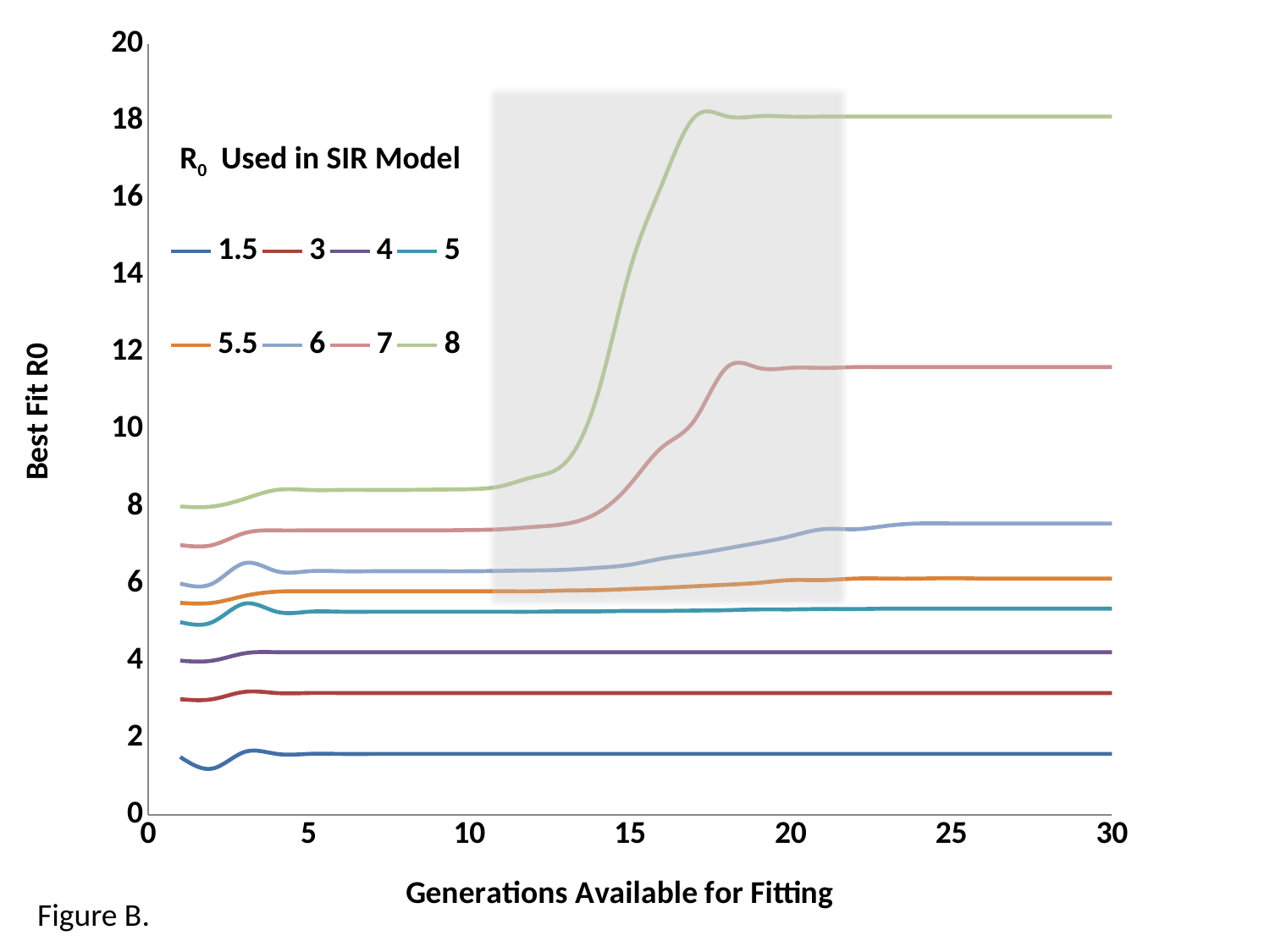

### Chart
| Category | 1.5 | 3 | 4 | 5 | 5.5 | 6 | 7 | 8 |
|---|---|---|---|---|---|---|---|---|
R0 Used in SIR Model
Figure B.

## Slide 3
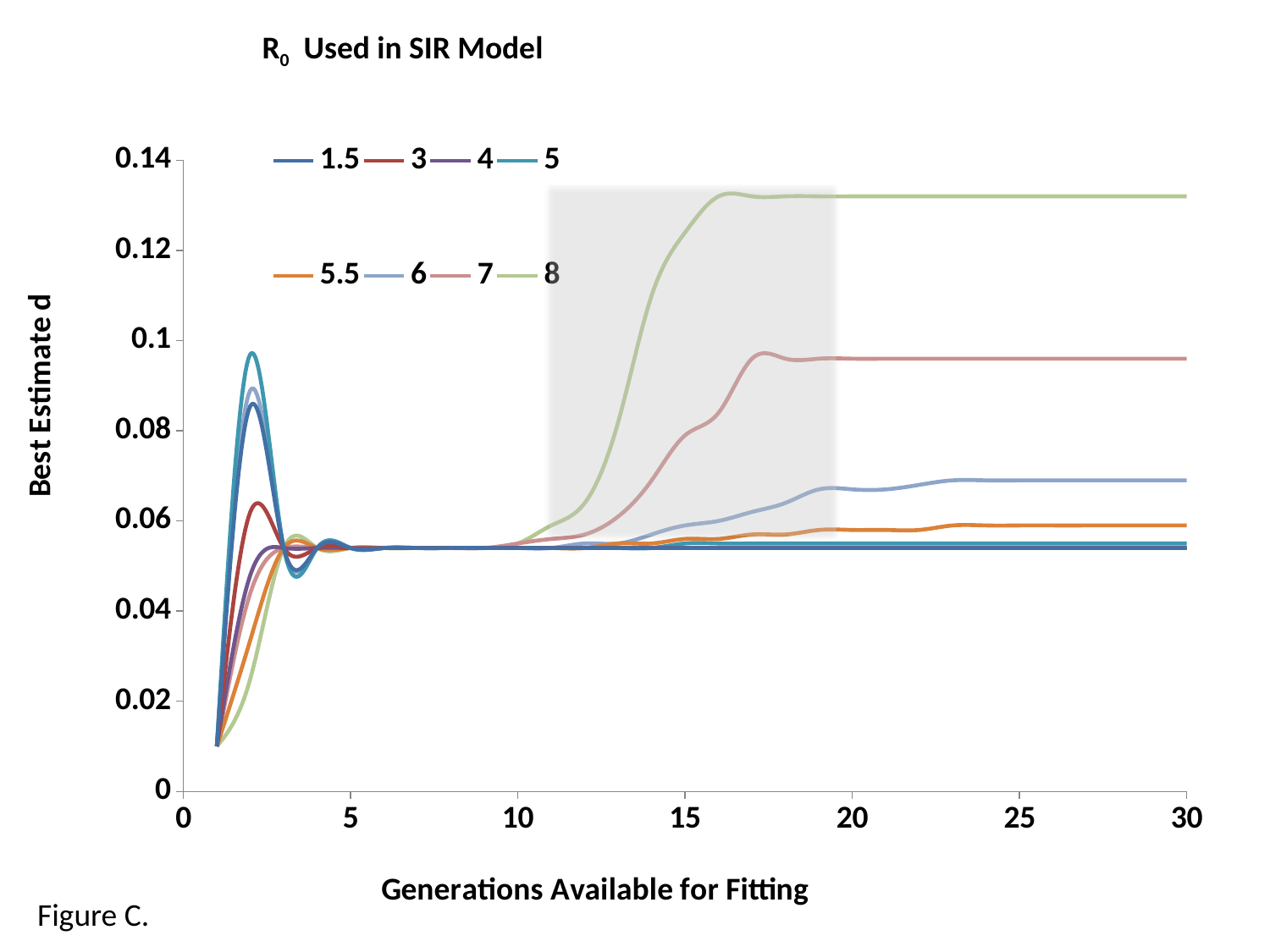

### Chart
| Category | 1.5 | 3 | 4 | 5 | 5.5 | 6 | 7 | 8 |
|---|---|---|---|---|---|---|---|---|R0 Used in SIR Model
Figure C.

## Slide 4
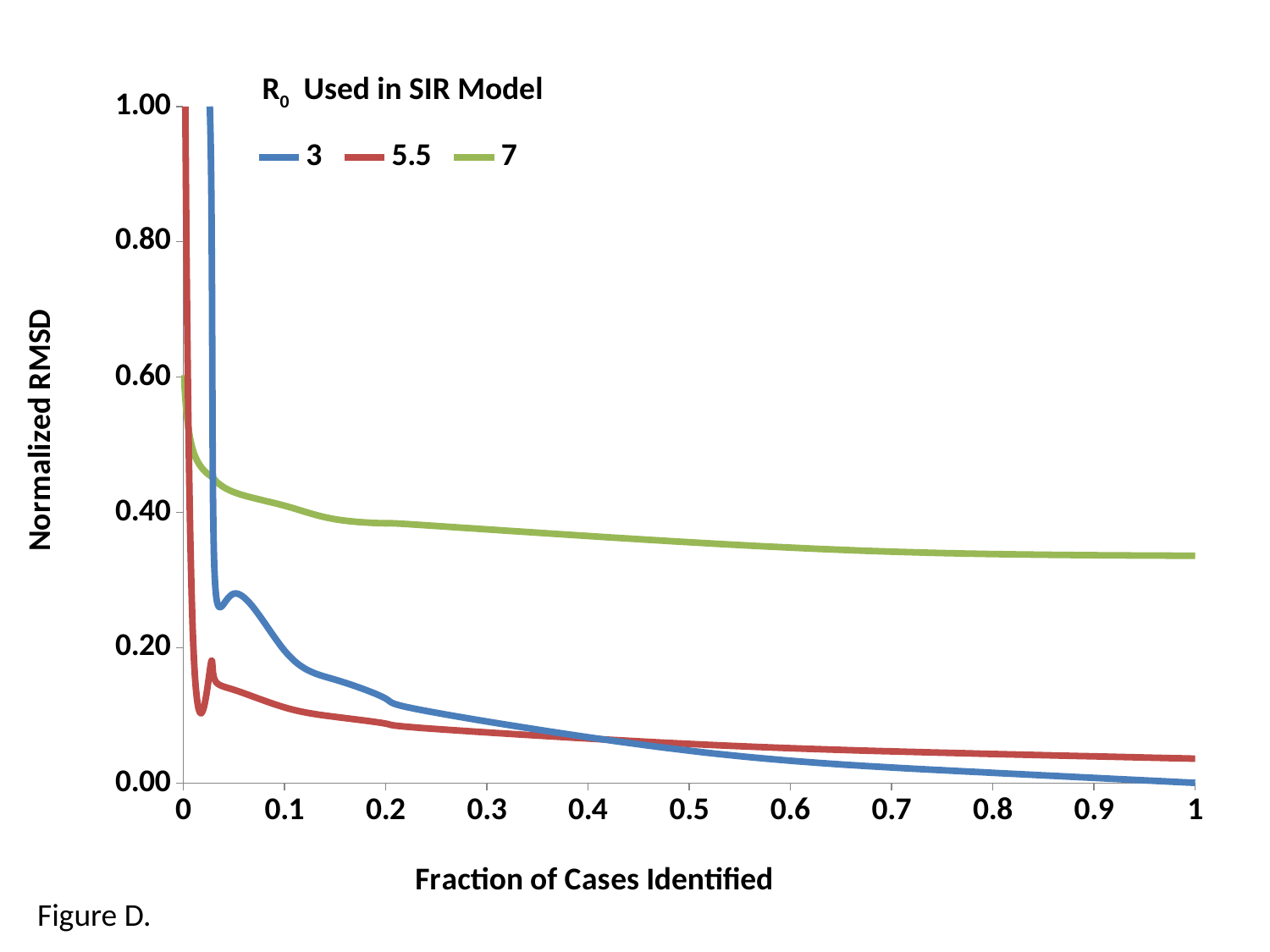

### Chart
| Category | 3 | 5.5 | 7 |
|---|---|---|---|R0 Used in SIR Model
Figure D.

## Slide 5
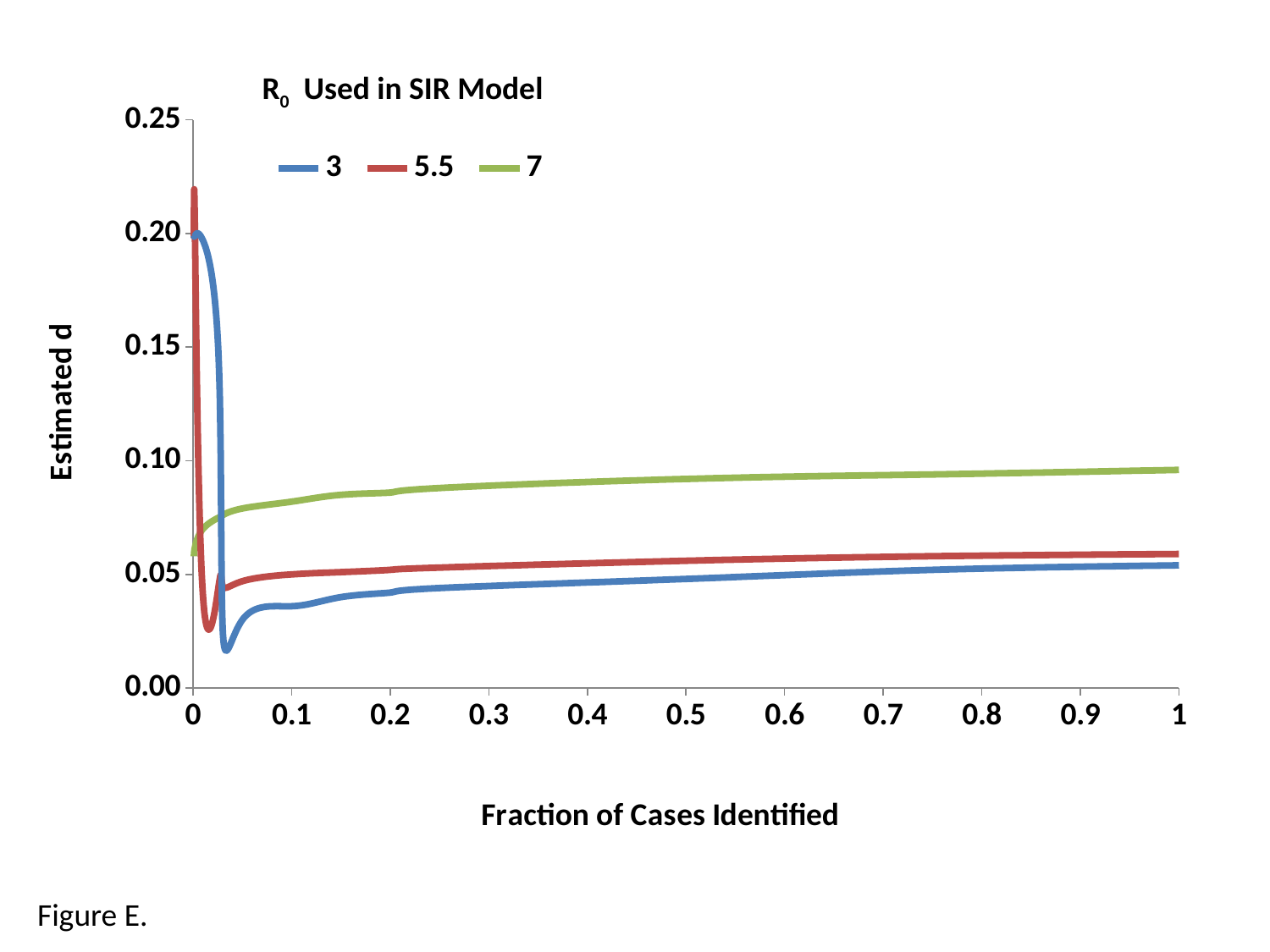

### Chart
| Category | 3 | 5.5 | 7 |
|---|---|---|---|R0 Used in SIR Model
Figure E.

## Slide 6
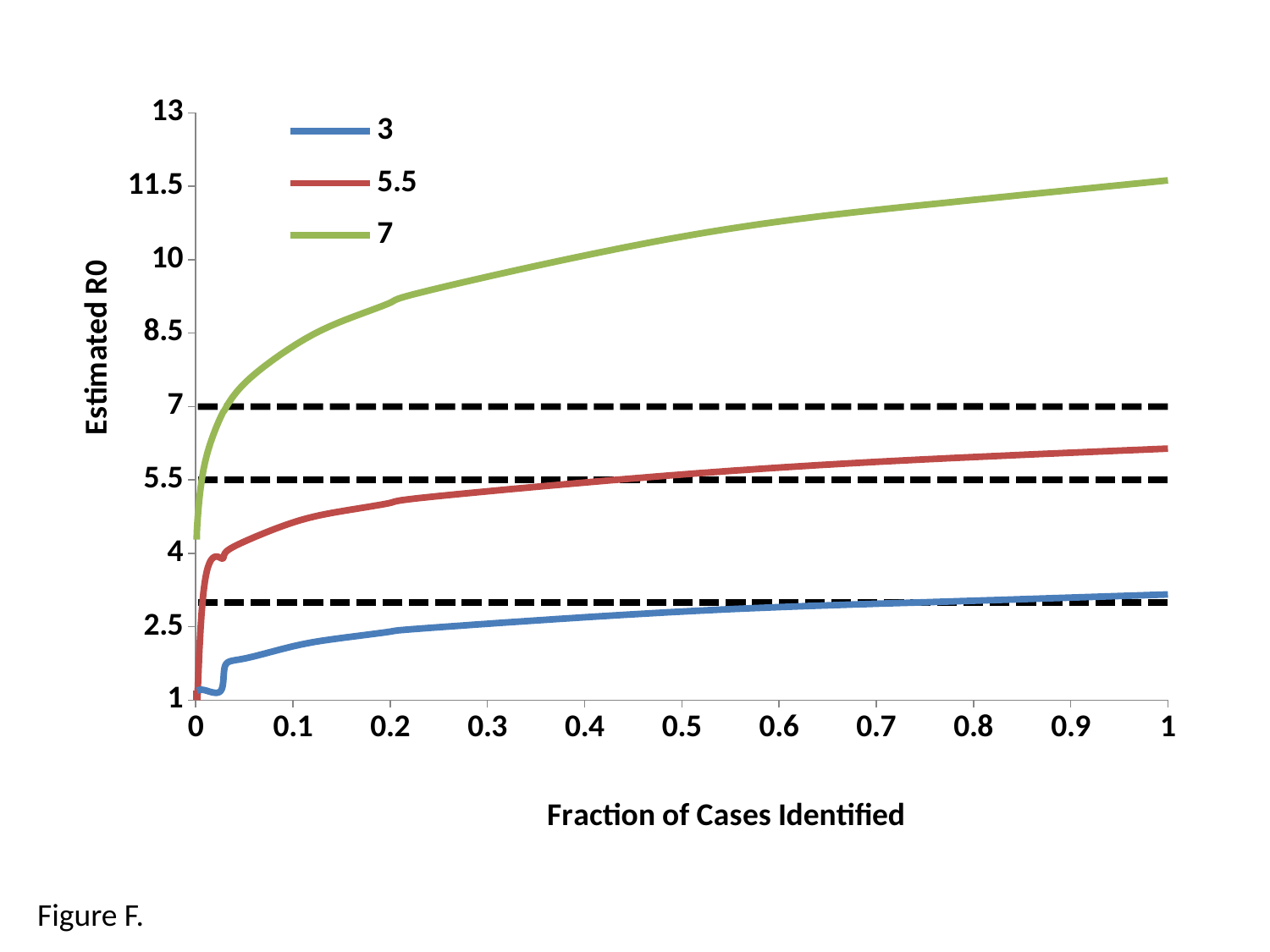

### Chart
| Category | 3 | 5.5 | 7 | | | |
|---|---|---|---|---|---|---|Figure F.

## Slide 7
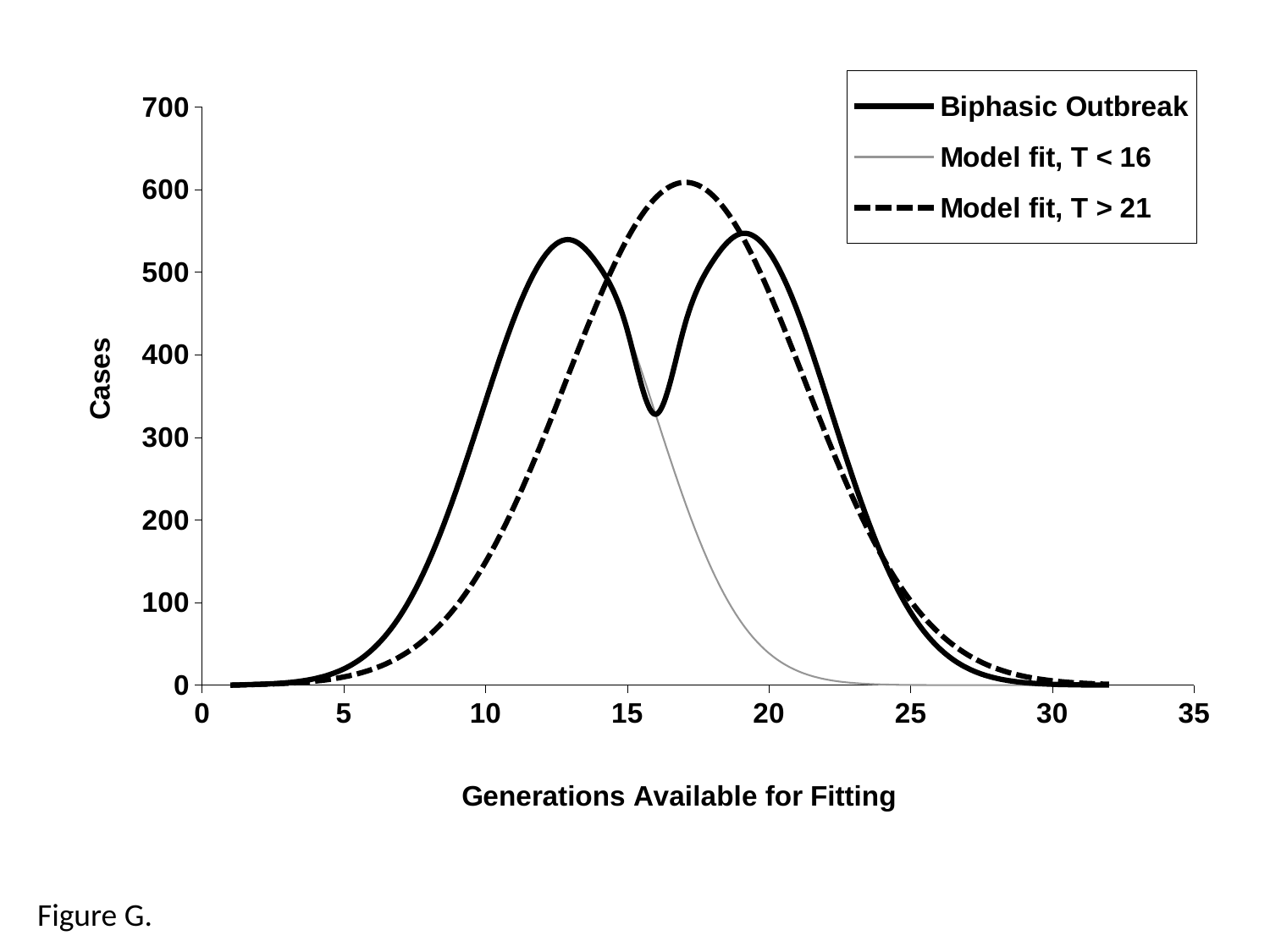

### Chart
| Category | | | |
|---|---|---|---|Figure G.

## Slide 8
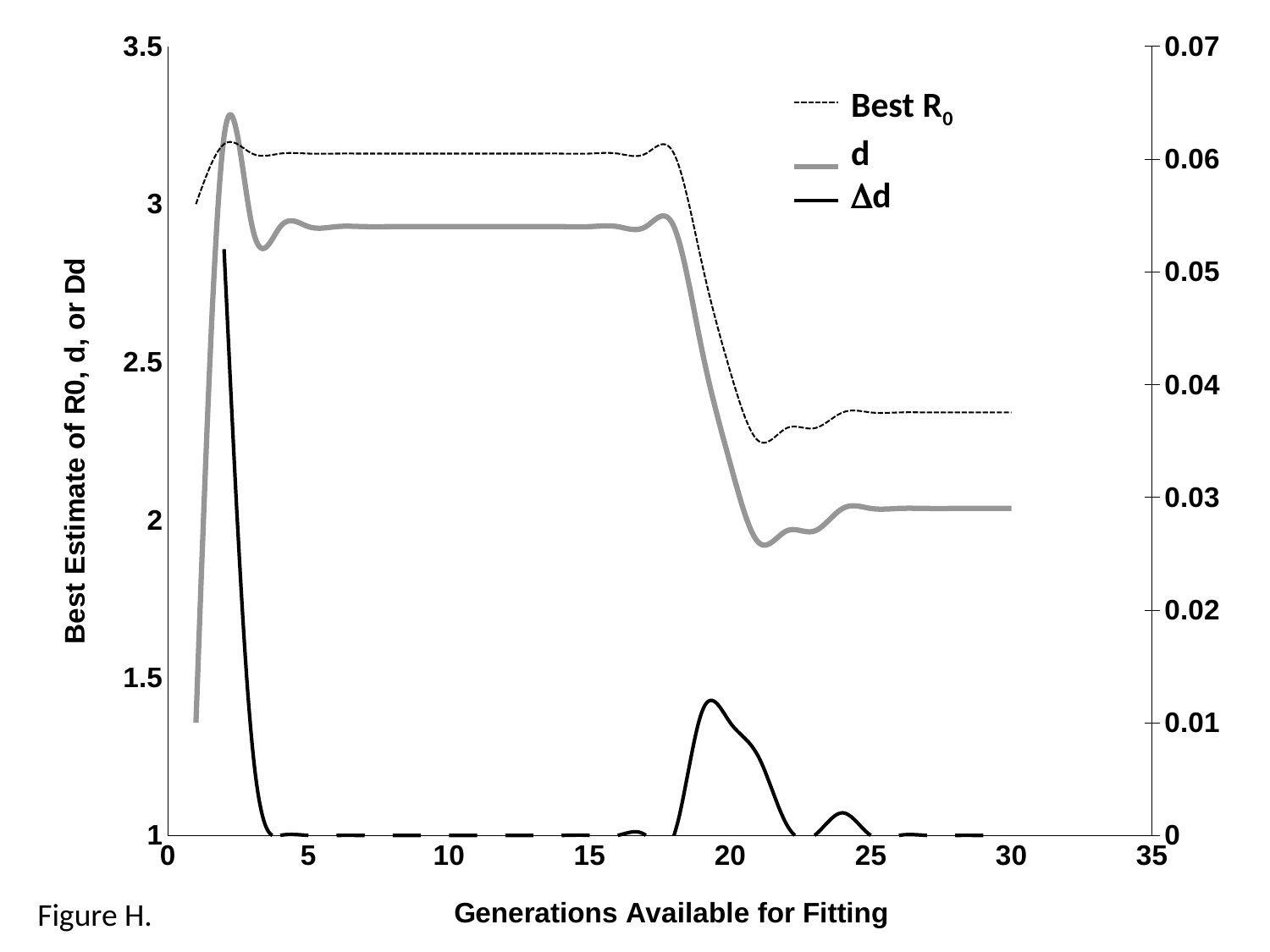

### Chart
| Category | Best R0 | d | Dd |
|---|---|---|---|Best R0
d
Dd
Figure H.
